# Sex matters: the frequently overlooked importance of considering sex in computational models

**DOI:** 10.3389/fphys.2023.1186646

**Published:** 2023-07-13

**Authors:** K. S. Burrowes, M. Ruppage, A. Lowry, D. Zhao

**Affiliations:** ^1^ Auckland Bioengineering Institute, University of Auckland, Auckland, New Zealand; ^2^ Department of Nursing, Faculty of Medical and Health Sciences, University of Auckland, Auckland, New Zealand

**Keywords:** personalised medicine, physiological models, sex-specific, sex differences, healthcare, digital twin

## Abstract

Personalised medicine and the development of a virtual human or a digital twin comprises visions of the future of medicine. To realise these innovations, an understanding of the biology and physiology of all people are required if we wish to apply these technologies at a population level. Sex differences in health and biology is one aspect that has frequently been overlooked, with young white males being seen as the “average” human being. This has not been helped by the lack of inclusion of female cells and animals in biomedical research and preclinical studies or the historic exclusion, and still low in proportion, of women in clinical trials. However, there are many known differences in health between the sexes across all scales of biology which can manifest in differences in susceptibility to diseases, symptoms in a given disease, and outcomes to a given treatment. Neglecting these important differences in the development of any health technologies could lead to adverse outcomes for both males and females. Here we highlight just some of the sex differences in the cardio-respiratory systems with the goal of raising awareness that these differences exist. We discuss modelling studies that have considered sex differences and touch on how and when to create sex-specific models. Scientific studies should ensure sex differences are included right from the study planning phase and results reported using sex as a biological variable. Computational models must have sex-specific versions to ensure a movement towards personalised medicine is realised.

## 1 Introduction

Personalised medicine is about tailoring healthcare to an individual. This approach relies on an understanding across the scales of biology–from genes and cells/tissue up to the organ and environmental levels. Personalised medicine is contrary to the one-size-fits-all approach typically used in healthcare. The future of these innovations sits with our ability to decipher the huge amounts of data able to be acquired from the human body. We can sequence the whole genome, we know a lot about proteins, cells and tissues, and rapid advancements in medical and wearable technologies are enabling measurements of an array of biometric data. But how can we understand this information and use it to improve health? Mathematics, computers, and engineering alongside clinical medicine play a significant role in answering this question. The development of personalised computational models, or a ‘digital twin’, is one approach towards personalised medicine (Kamel Boulos and Zhang, 2021; Armeni et al., 2022). These types of *in silico* models can be personalised to an individual using available clinical or genomic data, data from wearable sensors, or other forms of biological or physiological information. The advantages of these types of models is that they can be used to predict physiology in a simulated environment, for example, how a given intervention or treatment plan could impact on the individual. A complete digital twin still seems like a vision of the future, but vast strides are being made in this area as you will see within this special issue. A major challenge to overcome is gaining an understanding of the underlying biology for everyone, across different ethnicities, across sexes, and across different environmental exposures.

Sex differences in health and biology is one aspect that has frequently been overlooked, with young white males being seen as the “average” human being. This has not been helped by the lack of inclusion of female cells and animals in biomedical research and preclinical studies or the historic exclusion, and still low in proportion, of women in clinical trials. The lack of recognition of sex, and gender, differences in biology and human health is an issue that research is only beginning to rectify. It is no longer acceptable for basic science studies to be performed exclusively in male animals, or for women to be excluded from clinical trials. Sex differences in genetics, epigenetics (Novella et al., 2021), molecular biology (Wizemann and Pardue, 2001), immunology (Klein and Flanagan, 2016), drug metabolism (Soldin and Mattison, 2009), and anatomy (Dominelli et al., 2018; Ripoll et al., 2020; St. Pierre et al., 2022) exist. These differences lead to differences in susceptibility to various diseases, symptoms in a given disease, and outcomes to a given treatment. The COVID Pandemic has finally opened our eyes to the fact that health is driven by biology and gender. Significant sex and gender differences were observed in terms of susceptibility, symptoms, severity, and mortality with worse prognosis in males in the acute phase and females more affected by Long-COVID Syndrome (Pelà et al., 2022). This highlights the fact that a one-size-fits-all approach to health and disease is not fit for purpose. Neglecting these differences could lead to poor health outcomes for both men and women.

The terms “sex” and “gender” are often used interchangeably, but they are not the same (Torgrimson and Minson, 2005). Sex refers to biological attributes, including genetics and reproductive organs. Gender is shaped by social and cultural influences and may or may not align with an individual’s biological sex. While both sex and gender can influence health (Heidari et al., 2016), here we will focus on sex differences in the cardio-respiratory systems. There is an increasing body of literature around sex differences in biology, physiology, and health but more work is needed to close the sex and gender gap in health and healthcare.

While anatomical differences, at the macro scale, should already be accounted for in personalised computational models, several differences are frequently overlooked. In this review, we summarise some of the many differences in anatomy and physiology between the sexes, with the first step of raising awareness to a wider audience that these differences exist. We begin by summarising the disparate origins of sex differences then focus on the lungs and cardiovascular systems. We also aim to highlight why including these differences in computational modelling and any healthcare advancements is important, describe models that have begun to consider sex differences, and discuss how and when sex differences should be included in computational models. Finally, we discuss what is needed by researchers working in this field to ensure we one day resolve the sex bias in healthcare.

## 2 Origins of sex differences

**FIGURE 1:**
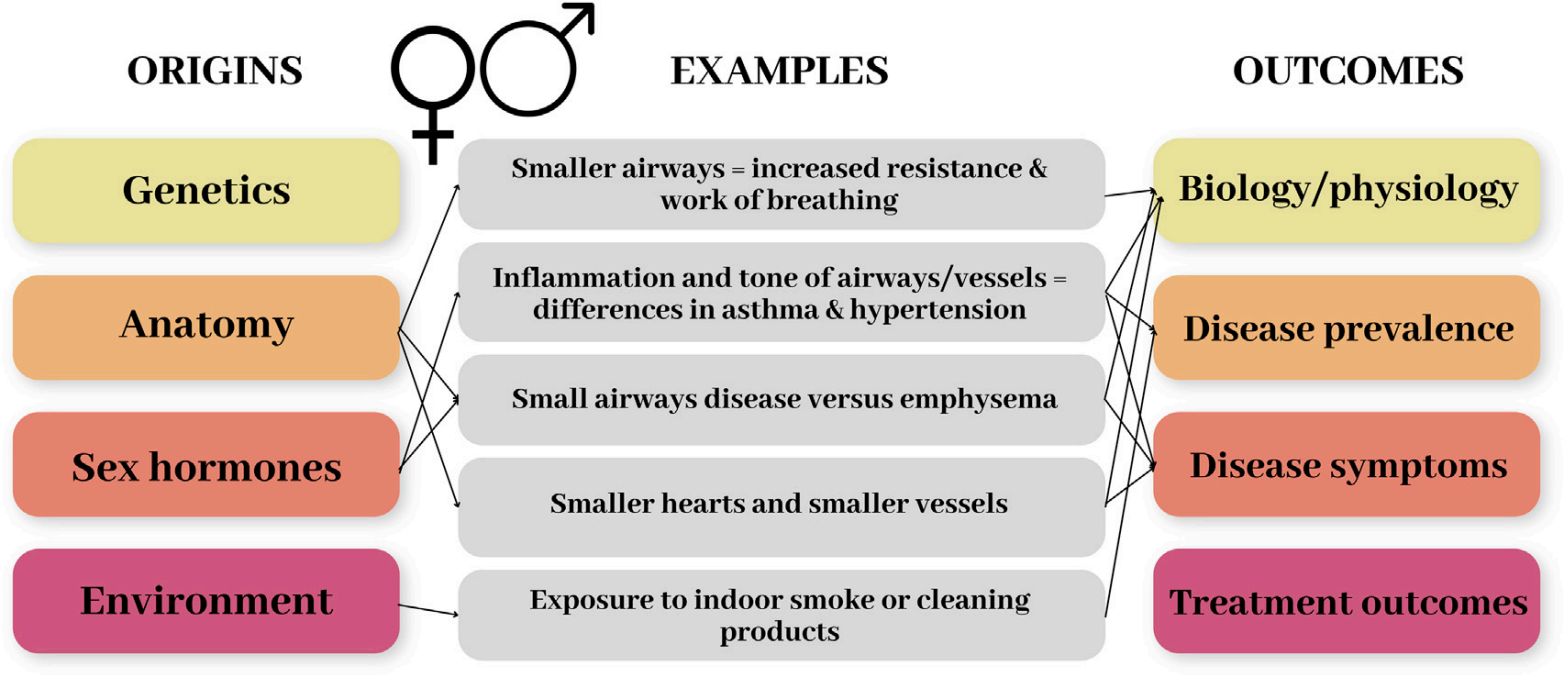
Schematic diagram demonstrating the origins of sex differences and the range of impacts/outcomes this can have. Some examples related to the respiratory and cardiovascular systems are included to demonstrate these.

Sex (and gender) differences in physiology and health can emerge as a result of several different factors. These include genetic and anatomical differences, hormonal – especially the sex hormones – differences, and for gender the environment or behaviours of an individual, such as domestic roles or cultural factors. Figure 1 demonstrates the origins of sex differences and the range of impacts/outcomes this can have, with some examples related to the respiratory and cardiovascular systems. In this review we will focus on anatomical and hormonal differences, and how these factors combine to produce differences in disease prevalence and outcomes.

### 2.1 Genetic differences

A detailed discussion of sex differences in genetics is outside of the scope of the current review. However, as an overview, men and women have very similar genomes, with the phenotypic differences across sexes being determined, initially, by genes on the sex chromosomes. These sex chromosomes comprise approximately 5 percent of the total human genome. The male genome differs from the female genome in the number of X chromosomes that it contains, as well as by the presence of a Y chromosome. A change in the activity of just a single gene can have a large effect on the organism that carries that gene, meaning that any sex differences – even one gene – can impact on physiology (Wizemann and Pardue, 2001). Sex differences in gene expression and co-expression have been studied using RNA-sequencing in different tissue types (Hartman et al., 2021; Khodursky et al., 2022). These studies have shown sex differences in gene expression associated with a range of important biological functions, including sex hormones and immune response as well as other signalling pathways (Khodursky et al., 2022). Differences in the basic cellular biochemistry of males and females can affect an individual’s health. However, understanding exactly how is complicated by the body’s holistic integrative function and the effect of sex on gene network biology is largely unknown.

### 2.2 Anatomical differences

While there is large variation in the size of humans and their internal organs at a population level, males are on average larger than females. This is applicable in humans and other large mammals (Touraille and Gouyon, 2008). On the whole, even when normalised for weight and height, females have comparatively smaller organs than males. But it is more than this. The proportions of organs can differ between males and females and the structure of the cells and tissue can differ giving rise to variations in function. We delve deeper into anatomical differences in our exemplar organs (the lungs and heart) in subsequent (Sections 3.1.1 and 3.2.1).

### 2.3 Sex hormones

Hormones are natural substances or chemicals that are produced in our bodies. Hormones can be described as chemical messengers. Sex hormones are vital in sexual development, reproduction, and in general health. These hormones are produced in the adrenal glands and the gonads. For females this is in the ovaries (at least until menopause), while for males it is in the testes. Males and females have the same sex hormones, the main ones being oestrogen, progesterone, and testosterone. What differs is their production sites, the relative concentration, and their interactions with different organs and systems. The levels of sex hormones in our body changes over time. For females especially, hormones fluctuate throughout a lifetime and approximately monthly during the reproductive years. The largest variations for women are during puberty, pregnancy, and during perimenopause to menopause. While males do exhibit variation in their sex hormones over a lifetime, these changes are less pronounced than in females (Decaroli and Rochira, 2017). Sex hormones have a wide and disparate impact on our bodies beyond reproduction, ranging from our brain function and mental health (McEwen and Milner, 2017), immune system function (Lombardo et al., 2021), cardiovascular function (Willemars et al., 2022), the musculoskeletal system (Hart, 2023), our perception of pain (Vincent and Tracey, 2008; Hart, 2023), metabolism and impact of drugs (also due to other factors such as body composition) (Soldin and Mattison, 2009), and much much more. In the following subsections, we will include some examples of the role of sex hormones in differences in physiology and disease.

## 3 Examples of sex differences in biology and health

Without the knowledge that a problem exists, it is difficult to solve. Here we aim to raise awareness and provide some examples of known sex differences in biology and physiology. However, this is far from the full picture as there is a growing body of literature in this field. We focus on the respiratory and cardiovascular systems where we are aware of advanced computational modelling in the field.

### 3.1 Respiratory system

**FIGURE 2:**
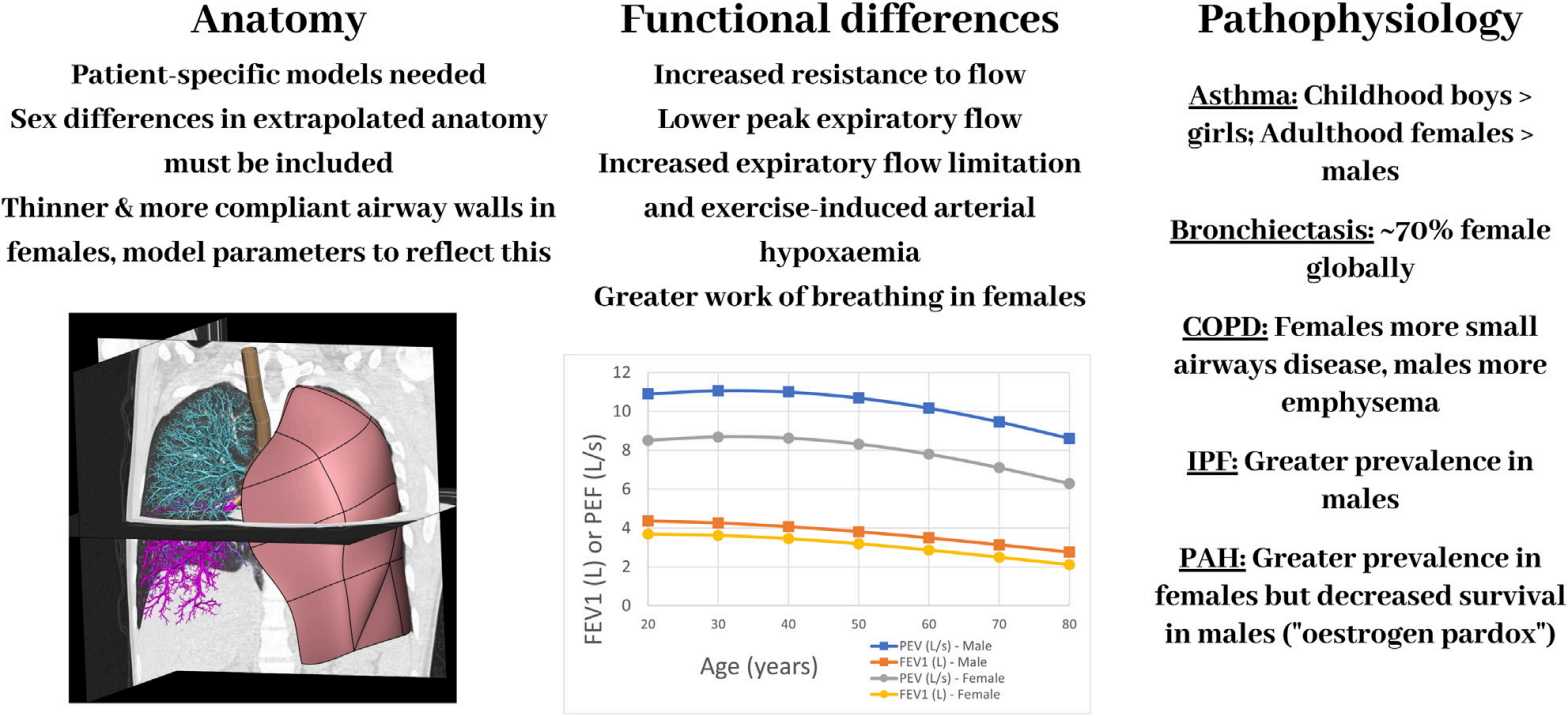
A schematic diagram showing some of the main differences between the female and male respiratory systems–in anatomy, function, and pathophysiology–and important aspects to consider for computational models. Image in left panel is a computational model of the lungs set within a CT scan. COPD: Chronic Obstructive Pulmonary Disease; IPF: Idiopathic Pulmonary Fibrosis, PAH: Pulmonary Arterial Hypertension. The plot of FEV1 (forced expiratory volume in 1 s) and PEF (peak expiratory flow) was created using equations to calculate mean values for males and females based on spirometry in 8,684 health, never-smoking adults from Kuster et al. (2008).

The main function of the respiratory system is gas exchange. Efficient gas exchange, and disruption of this, can be attributed to the matching of air to blood and the ability of oxygen and carbon dioxide to diffuse across the thin alveolar-capillary membrane. The lungs can be challenging to study experimentally due to the negative pressure they operate under normally *in vivo*. In the last 2 decades there have been substantial developments in computational modelling of the respiratory system, especially in terms of creating models that are realistic, in that they are more anatomically accurate and biophysically based. Advancements in imaging and computational technologies now enable personalised or subject-specific geometric models to be developed, representing the 3D branching structure of the airways and pulmonary vasculature. There are several examples in the literature (Werner et al., 2009; Kheyfets et al., 2015; Roth et al., 2017; Ashworth et al., 2023). However, to date there has been little application of computational models to assess sex-based differences in lung (dys)function. Here we summarise some of the key differences in anatomy, function, and disease between males and females motivating the need to consider these in future iterations of models and their clinical applications, demonstrated diagrammatically in Figure 2.

#### 3.1.1 Anatomical differences

**FIGURE 3:**
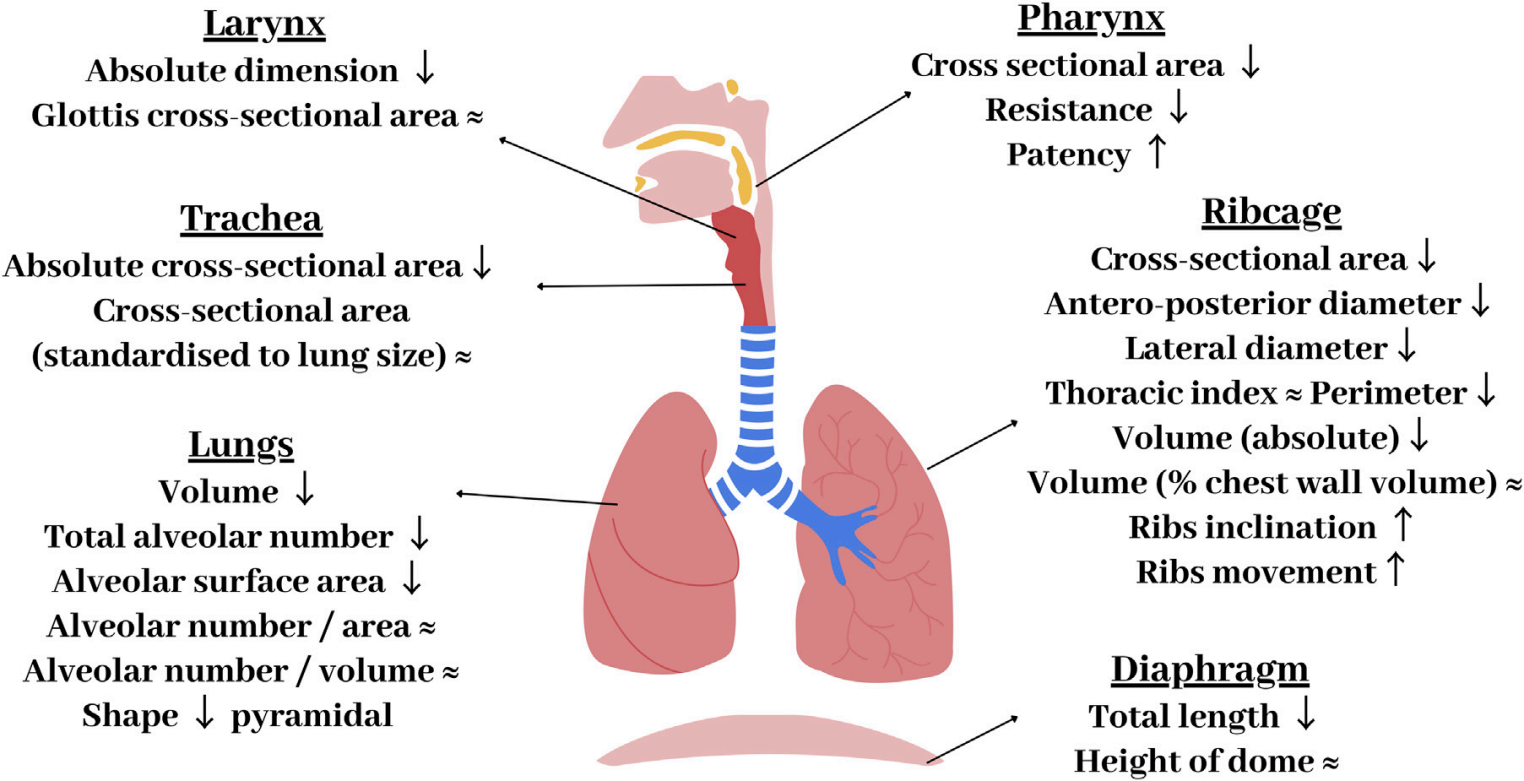
Summary of key anatomical differences of the female respiratory system compared with males. Arrows represent how female components differ to males: ↑ - increased in women compared to men, ↓ - decreased compared to men, ≈ - no differences between sexes.

The respiratory system is comprised of the lungs, airways, pulmonary circulation, ribcage, and the respiratory muscles. Whilst the overall anatomy can appear similar between the sexes, there are a number of variations which can be found in the respiratory system. Figure 3 summarises the differences in the anatomy of the airways, lungs, chest wall, and diaphragm between males and females. The most obvious difference is that females have smaller lungs than males. This is attenuated, but still persistent, when matched for height (Schwartz et al., 1988). Males have been found to have lungs that are ∼10–12% larger than females (Bellemare et al., 2003). This has been observed through various methods and studies, including morphometric and computed tomography (CT) studies (Bellemare et al., 2003; Torres-Tamayo et al., 2018). In attempts to make relative comparisons, male and female lungs have been compared for similar heights (Schwartz et al., 1988), lung size (Sheel et al., 2009; Molgat-seon et al., 2018), and lung capacity (Kim and Hu, 1998); however, male lungs remained relatively larger across all comparisons (Schwartz et al., 1988). Despite this size difference, males and females have the same number of alveoli per unit-area and unit-volume, with alveoli of similar dimensions (Lomauro and Aliverti, 2018). However, as males have larger lungs, they have a greater total number of alveoli and hence alveolar surface area for a given age and height (Thurlbeck, 1982).

Overall, males have significantly longer airways than females, with the female airway lengths being ∼10%–14% smaller than males (Dominelli et al., 2018). Males also have larger cranial airways (Holton et al., 2014) and larger cross sectional-areas of the trachea and conducting airways (Sheel et al., 2009; Molgat-seon et al., 2018). A CT-based study by Dominelli et al. found that healthy adult females had central airways that were around 26%–35% smaller compared to males (Dominelli et al., 2018). These differences persist when matched for lung size (Sheel et al., 2009) and height (Dominelli et al., 2018). This discrepancy in the ratio of airway calibre to lung volume has been termed dysanapsis. Through the process of growing into an adult, dysanapsis occurs in females resulting in a disproportionate relationship between the size of lungs and airways (Merkus et al., 1993). This has functional implications due to the impact of luminal area on airflow resistance (Kaminsky, 2012) and may be responsible for the worse respiratory outcomes reported in woman (refer to Section 3.1.3). Large airway/vessel geometry can be extracted from CT scans and used to develop subject-specific models. However smaller airways/vessels (if included) need to be approximated in terms of their length and diameter. Including relevant sex-specific information around anatomy is important in determining realistic function.

The shape of the lungs, ribcage, and diaphragm also differ between the sexes, with females possessing a more “prismatic” lung geometry (Torres-Tamayo et al., 2018), a narrower ribcage (Bellemare et al., 2003), and a smaller and more dome shaped diaphragm (Lomauro and Aliverti, 2021). Differences in shape have been shown to have consequences for tissue mechanics and resultant mechanics of breathing; with a link between lung shape and tissue mechanics being previously demonstrated in a computational modelling study (Tawhai et al., 2009), indicating sex specific lung shapes are important to capture. It has been proposed that the female diaphragm is less susceptible to fatigue than men during intense exercise (Guenette et al., 2010). During pregnancy, the diaphragm is impeded due to the enlargement of the uterus. The angle of rib inclination also differs, with more angled ribs in women. This has been proposed to be mechanically beneficial, enabling greater muscular contributions to inspiratory pressure swings, affecting the resulting contractile force produced by the intercostal muscles (Ratnovsky and Elad, 2005).

#### 3.1.2 Functional differences

Differences in sex-based anatomy give rise to known (and likely several unknown) functional differences, including fluid flow rates, lower maximal respiratory pressures (Molgat-seon et al., 2018), the regulation of lung volume, pressure variation during breathing and the consequent work of breathing (Lomauro and Aliverti, 2021). For example, the smaller airways in female lungs result in lower peak expiratory flow and vital capacity (Molgat-seon et al., 2018). Differences are particularly pronounced during conditions of high ventilation rates, such as exercise. Studies have suggested that women are more susceptible to respiratory system limitations during exercise compared to men. In particular, females are more likely to experience expiratory flow limitation and exercise-induced arterial hypoxaemia, and have a higher metabolic cost of breathing for a given ventilation (Sheel et al., 2016; Molgat-Seon et al., 2018; Dominelli et al., 2019). Increased expiratory flow limitation is thought to be due to the fact that females have thinner airway walls compared to males. This coincides with suggestions that females have more compliant airways and are therefore more prone to collapsing than males, with Bhatt et al. finding that females had a greater prevalence of expiratory central airway collapse (7.2% vs. 3.1%) (Bhatt et al., 2016). This greater airway collapse has been associated with greater dyspnoea (breathlessness) and worse respiratory quality of life (Boiselle et al., 2013). Another functional difference between males and females is the work of breathing (WOB), with females having a greater WOB for a given ventilation, which may be particularly important during exercise (Guenette et al., 2007; Dominelli et al., 2015).

In terms of lung mechanics, breathing is facilitated by a combination of inspiratory ribcage muscles and contraction of the diaphragm (Lomauro and Aliverti, 2018). Whilst both of these mechanisms are utilised in males and females, their contributions towards breathing differ. It has been proposed that the inspiratory ribcage muscles contribute more in females than males (Bellemare et al., 2003). Differences in lung shape will no doubt influence the distribution of pressures within the lung tissue during breathing (Tawhai et al., 2009), but whether this impacts on overall lung function is unknown.

Sex hormones are important in lung inflammatory processes, breathing control, and in response to diseases. Hormones can influence airway tone and inflammation, affect different lung cell types, e.g., alveolar macrophages, neutrophils, dendritic cells and eosinophils (Townsend et al., 2012; Fuentes and Silveyra, 2018), and impact on the growth and resultant size of respiratory components. In terms of lung growth and airway calibres, dysanapsis is not noted in children <13 years old, with studies reporting no sex differences in cross-sectional areas (Kuo et al., 2018; Ripoll et al., 2020). As this is typically the age of puberty (Krieger et al., 2015), it supports the notion that airway diameter is affected by hormones. It has previously been concluded that prepubertal male boys have smaller airways than girls and that for the same total lung capacity boys had smaller flows than girls (Ripoll et al., 2020). During puberty, male airway size catches up and then overtakes that of females, indicating a higher growth rate in males (Merkus et al., 1993). These changes relate to the increased prevalence of asthma in males (pre-puberty) and females (post-puberty).

Hormones also impact on the tone of airways and blood vessels. A study examining the vasomotor effects of different hormones on rat pulmonary and coronary arteries found acute dose-dependent dilation of vessels with all hormones tested (17β-oestradiol–the predominant circulating oestrogen in women, testosterone, progesterone, and cortisol) (English et al., 2001; Kararigas, 2022). In pulmonary arteries, progesterone was found to have the greatest effect, followed by testosterone, cortisol, and oestrogen. In coronary arteries, the order was slightly different with testosterone having the greatest effect followed by progesterone, oestrogen, and cortisol. This suggests hormones are important in the aetiology of vascular diseases (English et al., 2001). Progesterone, and derivatives of this sex hormone, has also been found to inhibit airway smooth muscle contraction, suggesting a bronchodilating effect, in guinea pig tracheas (Montan and Perusquı, 1997; English et al., 2001). Testosterone is proposed to have a protective role in the airways as it causes bronchial tissue relaxation, reduces the response to histamines, and attenuates airway inflammation (Verma et al., 2011; Lomauro and Aliverti, 2021). This is in line with human studies showing that men with higher levels of testosterone had better lung function (Mohan et al., 2015; Lenoir et al., 2020). In contrast, oestrogen has been found to increase airway inflammation in the presence of allergens (Tam et al., 2011), contributing to higher prevalence of asthma in females after puberty. A randomised study found that early menopause significantly reduced the risk of airflow obstruction (van der Plaat et al., 2019), corroborating suggestions that a reduction in the female sex hormone has a protective effect on the airways. This is further evidenced by studies that have shown a ∼10% increase in asthma exacerbations during pregnancy (a time of increased oestrogen and progesterone) and as many as 40% of female asthma sufferers reporting premenstrual worsening of symptoms (Townsend et al., 2012). These findings highlight that the sex hormones can play an important role in the respiratory system and morbidities.

Another example of the impact of female sex hormones and their fluctuation over the menstrual cycle on lung physiology comes from a study by Behan and Kinkead (Behan and Kinkead, 2011). This study reported variations in female ventilatory response to hypoxia and hypercapnia depending on the phase of the menstrual cycle, indicating that fluctuations should be monitored (Behan and Kinkead, 2011). The impacts of changing sex hormones over the menstrual cycle on lung physiology highlights the complexity and importance of ensuring more females participate in research and clinical studies. In addition, it is vital that the stage of the menstrual cycle during measurements are recorded or standardised. These differences indicate that not only anatomy needs to be sex-specific in the development of computational models, but also function and cell-level responses should be adapted for male and female models when applicable.

#### 3.1.3 Pathophysiology

Lung disease has a global prevalence of approximately 544.9 million people and is the third leading cause of death worldwide (Gargaglioni et al., 2019; Somayaji and Chalmers, 2022). The three most common respiratory disorders are: Asthma (334 million people, 1 in 7 children), sleep-disordered breathing (100 million people), and Chronic obstructive pulmonary disease (COPD) (65 million people) (Gargaglioni et al., 2019). Interestingly, all three of these diseases share a common factor, that of being sexually dimorphic. While both men and women are at risk of respiratory disease, there are several key sex differences in disease prevalence, disease presentation, disease progression, and treatment outcomes. There are also differences in the immune response between sexes. Females have a more potent inflammatory immune response (Chrousos, 2010), with female lymphocytes and monocytes having greater immune-inflammatory reactivity (Cutolo et al., 2009). This plays a role in the prevalence of diseases that have an inflammatory aspect to their aetiology. These differences in disease prevalence and pathophysiology can arise from both anatomical and/or hormonal differences. Understanding these differences is crucial for effective diagnosis and treatment. Here we will highlight some of these differences with a focus on COPD, asthma, and pulmonary hypertension.

##### 3.1.3.1 Chronic obstructive pulmonary disease (COPD)

COPD is a chronic lung disease which causes airflow obstruction in the lungs. COPD encompasses a spectrum of diseases, with emphysema and chronic bronchitis being the two most common conditions. However, many individuals present a combination of these two conditions (Kim and Criner, 2013). Smoking and environmental pollution are the greatest risk factors for COPD. Historically, COPD has been considered a male dominated disease due to higher smoking rates in men. However, this trend is changing with the smoking gap between the sexes narrowing, and consequently COPD is now a critical health issue for females too (Ntritsos et al., 2018)

Sex differences have been proposed around the effect of smoking or pollution inhalation. Exact causes are unknown, but experimental studies suggest anatomical and sex hormones may be involved. One study, supporting the sex hormone hypothesis, studied the effects of 6-month chronic cigarette exposure in mice (Tam et al., 2016). It was found that male and female mice responded differently to this exposure, with females developing small airway disease and airflow obstruction, whereas male mice, for the same exposure, developed more emphysema. However, in female mice which had a reduction in female sex hormones (*via* ovariectomy or tamoxifen, an oestrogen receptor blocker), there was an increased level of emphysema development, presenting a trend similar to that seen in the male mouse population (Tam et al., 2016). In humans, it was shown that for a given exposure, female smokers (>45–50 years old) had a more rapid decline in lung function (Gan et al., 2006), and a more rapid progression in CT lung density when compared to male smokers (Coxson et al., 2013). The influence of anatomical (and functional) differences relating to this could be the deposition of particles in the lungs. Kim et al. showed a marked difference in regional deposition patterns between males and females, with an increased deposition in the proximal airways in females (Kim and Hu, 1998). Additionally, it was found that females had a higher deposition of fine particle matter <2.5 µm in lung airways (Shin et al., 2022). This could be due to female lungs being smaller, meaning that the “dose” from each cigarette is relatively more concentrated when compared to larger male lungs (Lomauro and Aliverti, 2021). This has been demonstrated in a computational modelling study. Poorbahrami et al. used a full lung *in silico* model to simulate particle transport in different sized lungs representative of an infant, child, and adult anatomy (Poorbahrami et al., 2021). They found increased deposition fraction and higher tissue concentrations (of inhaled particles) when simulating smaller lungs due to the smaller airway diameters and resultant increases in velocity. This may have important implications both in inhaling harmful particles and in dosage for inhaled medications. While the size differences between male and female lungs will be smaller, these differences may still contribute to increased deposition in female lungs. A final hypothesis is that females metabolise the chemicals found in cigarettes differently. For example, one study showed a greater susceptibility to polycyclic aromatic hydrocarbons amongst females (Uppstad et al., 2011).

Another aspect around sex differences in COPD prevalence relates to diagnostic bias. COPD is still considered a “men’s disease” despite the recent changes in propensity. This association has led to the under-diagnosis of COPD in females (Perez et al., 2020). An example of the bias in data is the widespread use of the Fletcher-Peto curve of forced expiratory volume in 1 s (FEV_1_) decline in smokers (Somayaji and Chalmers, 2022). This curve, first published in 1977, is derived entirely from studies of males (Fletcher and Peto, 1977). Other male-dominated studies have followed, for example, the BODE (body mass index, airflow obstruction, dyspnoea, exercise capacity) cohort study by Casanova et al. (Casanova et al., 2011) with data collection spanning from 1997 to 2009, in which 92% of participants were male. This creates challenges with understanding or identifying sex differences in smoking-related (or other) lung decline (Miller et al., 2014).

Setting the effects of cigarette smoking to the side, the percentage of the non-smoking COPD population is large and growing, with reports finding that never-smoking COPD accounts for about half of all COPD cases worldwide (Salvi and Barnes, 2009). Of the never-smoking COPD population, females account for more than two-thirds with moderate to severe airway obstruction (Lamprecht et al., 2011). It has been proposed that a difference in exposure to harmful gases may be influenced by societal norms, activities, and occupations. Traditional societal norms tends to see women spending longer indoors (Matz et al., 2014) and doing a greater proportion of household cleaning and cooking (Ly and Jena, 2018). These time frames lead to a greater exposure to indoor air pollution and fine particles from cleaning products or cooking (Lim et al., 2012). However, these exposures vary between socio-economical situations. For example, the prevalence of COPD is two to three times higher in rural women compared to urban women (Ekici et al., 2005). This has been correlated to chronic exposure to indoor smoke from biomass fuels burning, e.g., burning wood, charcoal, and other organic materials (Bruce et al., 2004) used as a source of heat for cooking or warmth (World Health Organization, 2014). The smoke from these biomass fuels has been found to increase respiratory disease by impairing lung function in the smaller airways (Raj, 2014).

##### 3.1.3.2 Asthma

Asthma is a chronic inflammatory airway disease that disturbs the respiratory tract, resulting in airway obstruction and bronchial hyper-responsiveness (Berge et al., 2013). Amongst children, <18 years, asthma has a higher propensity and increased severity among males, however this trend is found to be reversed in adults (Fuseini and Newcomb, 2017; Chowdhury et al., 2021). Significant shifts in prevalence and severity appears to overlap with events of major changes in hormone levels (as mentioned in Section 3.1.2), during pregnancy and the premenstrual phase, suggesting that sex hormones play a significant role in pathogenesis of asthma (Kynyk et al., 2011; Cephus et al., 2017). Additionally, an early menarche is associated with a greater risk of asthma and asthma prevalence while menopause appears to decrease the risk (Castro-rodriguez, 2015). Whilst menopause is reported to reduce asthma prevalence (Kynyk et al., 2011), evidence is lacking to suggest that menopause results in the reduction of asthma symptoms. In fact, one study has reported that during menopause, asthma symptoms may be increased (Real et al., 2008). Investigations into the impact of oral contraceptives on asthma symptoms reported a decreased risk of severe asthma while using such contraception (Nwaru et al., 2021).

Whilst all previously mentioned points suggest a deleterious effect from female sex hormones, the impact of male sex hormones remains unclear (Somayaji and Chalmers, 2022). However, some suggestions are that testosterone may have a protective role, supporting a balance between autoimmunity and protective immunity by preserving the number of regulatory cells (Canguven and Albayrak, 2011). Furthermore, it has been observed that males who report more severe or moderate asthma symptoms have testosterone levels lower than those who report mild symptoms (Gargaglioni et al., 2019).

##### 3.1.3.3 Pulmonary arterial hypertension

Pulmonary arterial hypertension (PAH) is characterised by high blood pressure that affects the blood vessels in the lungs (Batton et al., 2018). According to patient registries, there is a greater susceptibility for females to have PAH, with a female to male ratio of 1.4:1 in the UK. According to the European COMPERA registry, the global female/male ratio was 1.6:1. However, when separating the younger from the older population, <65 and >65, the ratios were distinctly different, being 2.3:1 and 1.2:1, respectively (Hoeper et al., 2013), illustrating that sex disparity attenuated with age. Hormonal changes after menopause may be related to this attenuation (Ventetuolo et al., 2014). Conversely, whilst females have a greater propensity for PAH, registries consistently reveal that females also have a better survival rate than males, regardless of age (Benza et al., 2010; Olsson et al., 2014).

The combination of these sex differences in PAH summarises the “oestrogen paradox”, that is, the supposed deleterious and protective effects of oestrogen. Females have been observed to be more susceptible, however, to also have better prognoses once afflicted with PAH. Tofovic et al. (Tofovic and Jackson, 2020) proposes a concept called the “three-tier concept”, which views estradiol as: 1) the protector of heathy pulmonary vasculature; 2) the instigator and perpetuator of disease in injured pulmonary vascular; and 3) the protector of the overloaded right ventricle, to explain this paradox.

### 3.2 Cardiovascular system

The cardiovascular system consists of the heart and the blood vessels (arteries, veins, and capillaries), which are collectively responsible for blood circulation to provide oxygen and nutrient supply to organs, tissues, and cells throughout the body. Cardiovascular diseases (CVDs), comprising a group of disorders of the heart and blood vessels, remain the leading cause of death worldwide for both women and men (Roth et al., 2018; Peters et al., 2019). Nevertheless, substantial differences exist in the prevalence and burden of various CVDs when stratified by sex, with a growing body of literature around sex-differences in the cardiovascular system (Beale et al., 2018; Gao et al., 2019; Peters et al., 2019; Reue and Wiese, 2022; St. Pierre et al., 2022). Differences in male and female cardiovascular physiology can be attributed to genetic, hormonal, environmental, behavioural and lifestyle factors, which are dynamic and interact throughout the life cycle (Molina and DiMaio, 2015). Having recognised such differences, major organisations such as the American College of Cardiology, American Heart Association, and European Society of Cardiology have released sex-specific normative ranges and clinical guidelines for the diagnosis and management of disease (Mosca et al., 2011b; Regitz-Zagrosek et al., 2018; Leslie et al., 2020; Perrino et al., 2021; Visseren et al., 2021). Despite these efforts, a recent commission found that there has been stagnation in the reduction of overall CVD burden for women, an area which remains understudied, under-recognised, underdiagnosed, and undertreated (Vogel et al., 2021).

Alongside ongoing research to better understand the underlying mechanisms of CVDs, there has been extensive work in the field of cardiovascular modelling, with advancements towards subject-specific heart models (Gray and Pathmanathan, 2018; Niederer et al., 2019), as well as whole-body circulation models (Hose et al., 2019). However, the majority of these models do not currently include sex-specific inputs, with only a few exceptions (Yang et al., 2012; Ahmed and Layton, 2020; Fogli Iseppe et al., 2021; Gonzalez-Martin et al., 2023). For the application of computational models to personalised medicine, it is essential to incorporate sex differences in order to devise optimal preventive and therapeutic strategies tailored to a particular individual. Furthermore, computational models provide a unique opportunity to study structure-function differences which could lead to better mechanistic understanding of the cardiovascular system by quantifying properties that are difficult or infeasible to measure *in vivo*. In this section, we will provide an overview of some of the important differences and implications in the consideration of developing *in silico* cardiovascular models.

#### 3.2.1 Anatomical differences

From an anatomical perspective, major differences exist between male and female cardiac and vascular morphology. On average, the human heart is reported to be the size of a fist, weighing approximately 300 g in adulthood (Gray, 1985). However, the heart is a dynamic organ that adapts its size and mass depending on our body’s demands throughout life. At birth, male and female hearts have roughly the same mass (∼20 g) but sex differences become statistically significant at puberty when left ventricular growth rate is significantly faster in boys than in girls. This is due to sex-specific hormonal influences that are imposed on the original anatomic pattern; this increases during adolescence and remains roughly constant during adulthood (De Simone et al., 1995). By adulthood, there are substantial differences in mass with female hearts, on average, being 26% lighter than male hearts (245 g female, compared to 331 g male) (St. Pierre et al., 2022). Therefore, it is important to note that the female heart is not simply a small, isometrically scaled down version of the male heart, with differences in the ratios between wall thickness and chamber size. Although scaling heart dimensions by lean body mass has been found to be effective, it can only help to reduce, but not eliminate, sex differences in cardiac geometry (St. Pierre et al., 2022). A detailed review of anatomical differences in standard clinical measurements between healthy males and females is presented in (Piro et al., 2010; St. Pierre et al., 2022).

As with cardiac remodelling, patterns of vascular aging (associated with increasing endothelial dysfunction and arterial stiffness (Lakatta, 2003)) also differ between males and females with implications for overt disease risk (Merz and Cheng, 2016). Men have greater endothelial dysfunction and arterial stiffness than women up to age 60, when age-related arterial dysfunction progresses at a faster rate in women. Additionally, postmenopausal women have stiffer arteries than males, even after accounting for body size and aortic diameter (Mitchell et al., 2004). While the findings of coronary artery disease due as a result of plaque formation are relatively consistent in men, these vary with age in women, where the prevalence of typical obstructive coronary disease is lower prior to menopause and increases substantially after age 50 (affecting only 14% of women below age 45 and up to 79% of women over the age of 75 (Pepine et al., 2015)).

#### 3.2.2 Functional differences

As several sex-dependent factors are involved in regulating cardiac function, it is important to account for their individual and interdependent roles. Sex differences have been found in blood pressure regulation (Ahmed and Layton, 2020), excitation-contraction coupling (Parks and Howlett, 2013), and contractility (Trexler et al., 2017), which subsequently produce differences in measured cardiac output, with female cardiac output around 22% lower than in males (St. Pierre et al., 2022). In terms of cardiac systolic function, ventricular ejection fraction increases with age in both sexes, with this increase being more pronounced in women (Gebhard et al., 2013). Both left and right ventricular stroke volumes are smaller in women (St. Pierre et al., 2022). Studies have also shown that the male heart exerts greater systolic blood kinetic energy, while the female heart exhibits significantly larger radial, circumferential, and longitudinal strains than the male heart for both left and right ventricular chambers of the heart (Kou et al., 2014; Lakatos et al., 2020).

At the cellular level, several sex-specific differences also exist in relation to constituents such as the extracellular and intracellular matrix, cardiac myocytes, endothelial cells, as well as cell distribution (Walker et al., 2021). Male cardiac myocytes contract more rapidly and with more force than female cardiac myocytes, likely as a result of differences in sarcomeric protein and calcium-handling function (Parks and Howlett, 2013). Although the number of cardiac myocytes between males and females is similar at birth (Senyo et al., 2014), female cardiac myocytes are less likely to undergo apoptotic cell death over time, supported by the finding that there is a significantly higher percentage of ventricular cardiac myocytes in female hearts than in male hearts at middle to early older age (Olivetti et al., 1995; Litviňuková et al., 2020). Nevertheless, cellular sex differences in the heart, caused by both sex hormones and chromosomal genotype, remains relatively understudied (Walker et al., 2021).

#### 3.2.3 Pathophysiology

Anatomical and physiological sex differences in the cardiovascular system ultimately influence the incidence and expression of CVDs (Shufelt et al., 2018), during which sex disparities become even more apparent. Due to the cardioprotective effect of sex hormones, namely, oestrogen, there are lower rates of CVD-related deaths in women (prior to menopause) (Woodward, 2019). As a result, CVD risk in women is often underestimated, alongside fewer diagnostic procedures and less aggressive treatment strategies (Gao et al., 2019). We must underline that, alongside traditional risk factors, sex-specific cardiovascular disease risk factors, such as obstetric and gynaecological history, including gestational hypertension, gestational diabetes, preterm delivery, premature menopause, and polycystic ovary syndrome, often underestimated, but also psychological, social, economic, and cultural factors, contribute to the global burden of cardiovascular disease in women (Vogel et al., 2021). A number of sex-specific differences in relation to prevalent conditions are discussed in the following subsections.

##### 3.2.3.1 Ventricular remodelling

Ventricular remodelling refers to ventricular adaptations – including changes in the geometry, mass, and volume – to physiological stimuli, including physical exercise, myocardial injury, or as a compensatory response to volume or pressure overload such as aortic insufficiency or aortic stenosis to maintain stroke volume. Four patterns of ventricular geometric adaptation can be identified by echocardiographically derived using left ventricular mass and relative wall thickness, the latter an index of geometry: normal geometry, concentric remodelling, concentric hypertrophy and eccentric hypertrophy (Ganau et al., 1992). Of the two patterns of left ventricular remodelling: concentric and eccentric geometry, the former is characterised by a greater increase in thicknesses compared to diameters and compared to the latter where diameters prevail over thicknesses. Sex has a profound impact on left ventricular remodelling, with women experiencing a more accelerated increase in ventricular wall thickness and concentric remodelling in response to different types of injures including aging, pressure and volume overload, and myocardial infarction or in the setting of risk factors such as hypertension and diabetes (Cheng et al., 2010; Piro et al., 2010). In these conditions women show a greater degree of left ventricular hypertrophy, increased relative wall thickness, smaller end-diastolic and -systolic volume, concentric remodelling and preserved systolic function compared to men that show a lower degree of left ventricular hypertrophy, more pronounced chamber dilation, eccentric remodelling and impaired systolic function (Piro et al., 2010).

##### 3.2.3.2 Cardiomyopathy

Cardiomyopathy is a collective term that refers to the disorders of the heart muscle that impairs cardiac output (Kåks et al., 2021). The term is generally used to indicate primitive cardiomyopathies such as dilated cardiomyopathy, hypertrophic cardiomyopathy, restrictive cardiomyopathy and arrhythmogenic cardiomyopathy (previously referred to as arrhythmogenic right ventricular cardiomyopathy), often genetically determined (Braunwald, 2017; Ciarambino et al., 2021). In 2006, the American Heart Association proposed the following definition “Cardiomyopathies are a heterogeneous group of diseases of the myocardium associated with mechanical and/or electric dysfunction that usually (but not invariably) exhibit inappropriate ventricular hypertrophy or dilatation and are due to a variety of causes that frequently are genetic. Cardiomyopathies either are confined to the heart or are part of generalised systemic disorders, which may lead to cardiovascular death or progressive heart failure-related disability” (Maron et al., 2006).

Current diagnostic criteria for cardiomyopathies are generally not sex-specific, despite intrinsic differences in normal anatomy (as discussed in Section 3.2.1 Anatomical differences). For example, the non-sex-specific maximum wall thickness threshold of 15 mm for hypertrophic cardiomyopathy implies that a female heart will have undergone a comparatively larger degree of hypertrophy than a male heart at the time of diagnosis (van Driel et al., 2019). Furthermore, the male:female diagnostic prevalence of dilated and hypertrophic cardiomyopathies is 3:1 and 3:2, respectively, indicating that women are less likely to be diagnosed with cardiomyopathy than men (Olivotto et al., 2005; Halliday et al., 2018; Cannatà et al., 2020). A further study found that sex-specific differences in left ventricular geometry, hyper-contractility, and diastolic function, contribute to a higher age-independent risk of diastolic heart failure in women with hypertrophic cardiomyopathy (Lu et al., 2020). Consequently, it is thought that implementing a multimodal computational framework that utilises patient history, medical imaging, and modelling, may provide a foundation for developing sex-based diagnostic criteria for cardiomyopathies (Lopez-Perez et al., 2015).

##### 3.2.3.3 Coronary heart diseases

For both women and men, coronary heart disease (also called ischaemic heart disease, caused by the narrowing of the coronary arteries as a result of atherosclerotic plaque) is the most significant contributor to CVD morbidity and mortality (Mosca et al., 2011a). As with cardiomyopathy, sex differences can be found in coronary heart disease, with females being more inclined to sustain vascular damage during myocardial infarction than males (Dedkov et al., 2016). Additionally, coronary heart diseases are linked to microvascular spasms and vasoconstriction in women, and to coronary occlusion and deposition of plaque in men (Franconi et al., 2017). Consequently, males adapt by growing coronary collateral vessels that bypass areas of occlusion, an event which is rare in females (Huxley and Kemp, 2018). Under ischaemic stress, females have been found to exhibit better resilience to oxygen deprivation than males owing to the cardioprotective effect of oestrogen in restoring mitochondrial activity and reducing apoptosis, thereby minimising infarct size (Dunlay and Roger, 2012; Wang et al., 2019). In addition spontaneous coronary artery dissection, a nonatherosclerotic aetiology of acute myocardial infarction, and Taktsubo stress cardiomyopathy are more common in women (Templin et al., 2015; Solinas et al., 2022).

The differences between sexes in mechanisms of disease progression, as well as in the subsequent adaptive response, have implications on clinical outcome. Therefore, the incorporation of sex-specific vascular networks into computational cardiovascular models may be able to provide additional insight into microvascular sexual dimorphism, with potential implications for sex-specific therapy and disease management.

##### 3.2.3.4 Hypertension

Hypertension (defined as an elevated blood pressure) is a global health issue and the greatest risk factor for mortality among all cases of CVD, despite various treatment efforts (Tsao et al., 2022). There are multiple complex pathophysiological mechanisms underlying hypertension, of which some are not fully understood. To date, a number of sex differences are known to be associated with blood pressure regulation, including the vasodilation of renal afferent and efferent arterioles (Zimmerman and Sullivan, 2013), renin-angiotensin system (Chappell, 2012), and renal sodium and fluid handling (Hu et al., 2019). Although there are differences in success rates between men and women, the prescription of antihypertensive therapy is typically not tailored to sex (James et al., 2014), with fewer women achieving blood pressure control compared to men (despite higher compliance in women (Gu et al., 2008)).

While there are only a few sex-specific computational models for blood pressure regulation (Leete and Layton, 2019; Ahmed and Layton, 2020), such models can provide insight into why males and females behave differently in hypertensive studies. Likewise, control over parameters of interest such as hydrostatic pressure, vascular smooth muscle tone, cellular interactions, would facilitate the investigation of sex-specific mechanisms in CVDs and hypertension (Lock et al., 2022).

##### 3.2.3.5 Heart failure

Although the overall incidence of heart failure is similar between women and men, sex differences are seen in specific phenotypes (i.e., heart failure with preserved ejection fraction (HFpEF) *versus* heart failure with reduced ejection fraction (HFrEF)). Data demonstrates that patients with HFpEF are more likely to be women, especially post-menopausal women (Sickinghe et al., 2019), obese, in advanced age, and/or with cardiac and non-cardiac comorbidities (i.e., hypertension, atrial fibrillation, anemia, depression, obstructive sleep apnea, diabetes and chronic kidney disease) (Xanthopoulos et al., 2018), than men (Beale et al., 2018). This indicates a role of estrogen, and the depletion thereof, in the development of HFpEF. In particular, steroid hormone 17β-estradiol (E2 oestrogen) plays a vital role in the cardiovascular system; regulating contractile function (micro)vascular function, metabolic processes, calcium signalling, gene expression and protein abundance. As E2 is involved in collagen regulation and collagen is responsible for vascular elasticity, a postmenopausal drop in E2 may contribute to reduced cardiac contractility and thereby onset of heart failure (Groepenhoff et al., 2020; Kararigas, 2022). There are several other sex differences that govern the incidence and prevalence of co-morbidities known to contribute to the pathophysiology of HFpEF. These include, but are not limited to: i) differences in ventricular structure, function, and patterns of remodelling, and metabolic inefficiency in women compared with men (Peterson et al., 2008); ii) differences in arterial elastance, higher pulse pressure, and aortic arch anatomy (Redfield et al., 2005), leading to alterations to the systemic and pulmonary circulation associated with increased arterial stiffness and impaired endothelial function (Paulus and Tschöpe, 2013); iii) differences in predispositions to hypertension, coronary heart disease, and atrial fibrillation (Beale et al., 2018); and iv) unique risk factors to women such as pregnancy (Keskin et al., 2017).

To date, the development of effective strategies for the prevention and management of HFpEF symptoms remains a challenge. Given the overrepresentation of women in the HFpEF population, there is scope for investigating the potential advantage of sex-specific approaches in the diagnosis and prognosis to improve outcomes in patients with heart failure. In this context, it is important to understand the influence of sex on the mechanisms associated with the relevant co-morbidities in order to optimise heart failure management in women.

## 4 Including sex differences in computational models

### 4.1 Consideration of sex in computational modelling to date

Considerations of sex differences within the field of computational modelling have been minimal to date. Those studies that have considered sex are rare and only evident in recent years. In 2020, Ahmed and Layton, developed the first sex-specific computational model of blood pressure regulation in the rat (Ahmed and Layton, 2020). The authors commented that despite well-known sexual dimorphism in blood pressure regulation, almost all previously published models were sex neutral. Their sex-specific models included sex differences in animal size (body weight and volume) and in functional parameters, such as cardiac output, filtration rates, and vascular resistance. Chen et al. (Chen et al., 2017) created a model of solute transport and oxygenation in the renal medulla of the kidney of a spontaneously hypertensive female rat. To their knowledge, every previously published computational model of rat kidney functions was based on the male rat. The model parameters in the female model were adjusted based on experimental data from female rats to account for differences in size and blood flow between the sexes. While this work is an important step forward, only some parameters in these models have been altered in a sex-specific way, with known differences not yet included.

Many modelling studies now create subject- or patient-based anatomical models which are applied in simulation studies. These models should, by default, include sex differences with respect to the detail derived from medical imaging or other patient information. This is once again progress in the right direction in terms of accounting for sex differences; however, if male and female models are lumped together rather than considered separately, sex differences will not be identified. For example, the modelling study of Foy et al. created 31 patient-based models derived from CT scans of both male and female participants (16 male; 15 female) (Foy et al., 2019). However, no consideration of sex was incorporated when analysing the simulation data, therefore it is not known whether these models demonstrated any sex differences in functional outcomes. Other studies, despite creating patient-based models, continue to focus on male participants only to exclude the confounding effects of any sex differences, such as (Ides et al., 2022). Finally, a study by Hedges et al. compared generic and subject-specific models when simulating forced expiration and included both male and female subject-based models derived from CT (Hedges et al., 2015). While this study did indicate results for discrete male and female-based models, no analysis or discussion of sex differences was included.

The organ that has received the most advanced analysis of sex differences *via* computational modelling is the heart. The work of Clancy and others provides some excellent examples (Yang et al., 2012; 2017; Fogli Iseppe et al., 2021). These computational studies have focused on understanding sex differences in cardiac electrophysiology and the risk of arrhythmias in males and females. They have employed a multiscale, systems biology approach, examining various biological scales such as gene expression, ion channel properties, and cell function. Their most recent study applied statistical learning to synthetic data, that was generated by simulating drug effects on cardiac myocyte models that captured male and female electrophysiology (Fogli Iseppe et al., 2021). This study specifically aimed to evaluate sex differences in the risk of drug-induced Torsade de Pointes, since female sex is a well-known risk factor for this condition. Torsade de Pointes (TdP), a rare but lethal ventricular arrhythmia, is a toxic side effect of many drugs. This work showed that male-biased predictive models consistently underestimate TdP risk in women (Fogli Iseppe et al., 2021). This study, and the group’s other work, exemplifies the importance of considering sex as a biological variable and the need for sex-specific models. Another study by Gonzalez-Martin et al. included the influence of both sex hormones and anatomical details (trabeculations and false tendons) on the electrophysiology of healthy human hearts (Gonzalez-Martin et al., 2023). These anatomically detailed models incorporated human myocyte models with sex phenotypes using the model of Yang and Clancy (Yang et al., 2012). This study demonstrated that neglecting either endocardial anatomical details or cell-level sex characteristics for cardiac electrophysiology simulations would lead to inaccurate predictions. Clear sex differences were noted, in agreement with physiology, for example, significant QT-interval increase and QRS duration in the detailed female human heart phenotypes.

### 4.2 How and when to incorporate sex in computational models?

**FIGURE 4:**
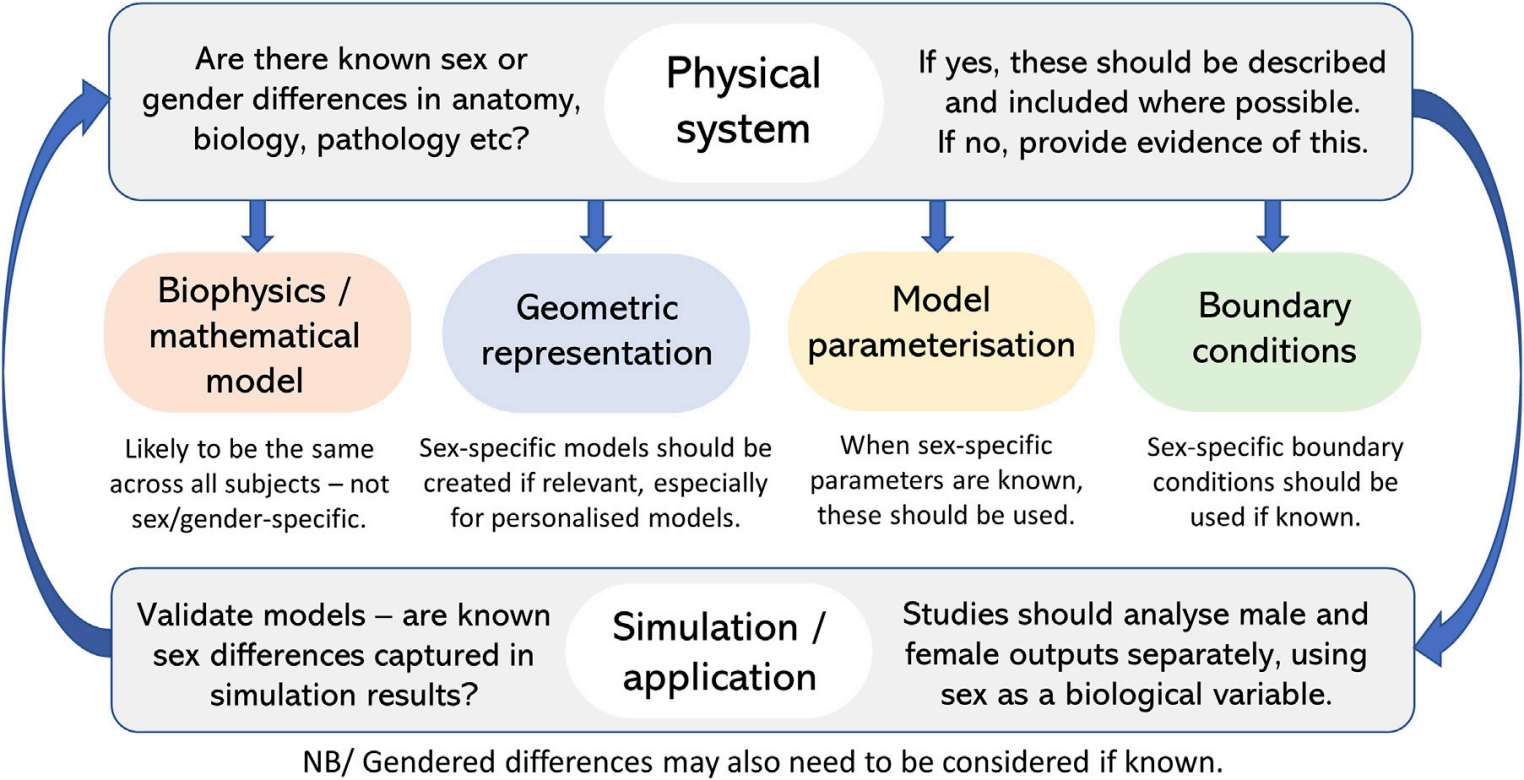
Checklist to ensure consideration and inclusion of sex (and gender) differences in model development and application where relevant.

With any modelling study, the detail required in a model is driven by the scientific question being posed. The examples provided above demonstrate a wide range of different modelling approaches–from simpler compartmental-type models, to subject-based anatomical models, to multiscale models including the link from organ to cell and beyond. These demonstrate that models, and the consideration of sex, may vary depending on the study. However, these studies all demonstrate the importance of including consideration of sex differences, where relevant, if we are aiming to develop models that are realistic and especially if aiming for personalised and patient-specific models. Figure 4 shows a checklist you can go through to ensure consideration of sex differences in model development and application.

One aspect that seems clear, is that sex-specific models are needed. Anatomical differences are likely the easiest to incorporate as these are well defined with modern model development that can, at least in part, be captured from medical imaging. Other considerations of sex can only be as good as the understanding of these differences in biology, physiology and pathophysiology. It seems evident that where sex differences are known to exist, models should acknowledge and include this. Models provide an excellent tool for incorporating these differences and assessing their impact on emergent function to further our understanding of differences.

As above, depending on the question being posed it may be enough to adjust parameters in a sex-specific way rather than having separate models for males and females. This may be the case for models with simplified, non-anatomical geometries. If patient-based anatomical models are being developed then models clearly need to be different for males and females, and in fact for each subject. In these cases, any known functional parameters that differ between sexes should also be included in these models. The beauty of computational models is that, as science advances and we continue to learn more about sex differences, models can be adapted and incremented to also include these novel factors. In the case where sex differences are unknown or not relevant, these should at the very least be discussed within the presentation of any modelling work.

## 5 Discussion

A big hurdle in narrowing the sex gap in healthcare is lack of awareness that such a gap still exists. Sex and gender perspectives in health and biology need to be integrated into all aspects of medicine, from health research to medical education, through to clinical practice. Here we have summarised only some of the many known sex differences in biology, physiology, and pathophysiology, as a way to increase awareness of these differences. No doubt there are still many differences yet to uncover. Here we have only scraped the surface; there are too many differences to describe. However, some of the examples illustrated here demonstrate how harmful neglecting these differences in any health-related research and practice can be, contributing to an ongoing lack of equity, affecting both males and females. Considerations of sex differences is important from the start of any research - during biomedical research through to the collection of human data. It is also vital in data analysis. If research fails to account for sex (and gender) during data analysis, there is a risk of harm through the assumption that study results apply to everyone. This can also lead to missed opportunities where potential differences may be found and exploited, leading to innovations and improvement in health outcomes. Many initiatives and Institutes have been formed around the world to address issues around sex/gender and health, with an excellent example being Canada’s Institute of Gender and Health, IGH (Canadian Institutes of Health Research, 2023). IGH’s mission is to foster research excellence regarding the influence of sex and gender on health, and to apply these findings to identify and address pressing health challenges facing men, women, girls, boys and gender-diverse individuals.

Guidelines and policies have been created around the world, and it is imperative that we are aware of these and aim to follow the proposed strategies. For example, the Sex and Gender Equity in Research (SAGER) guidelines were developed by a multidisciplinary group of academics, scientists, and journal editors by means of literature reviews, expert feedback, and public consultations at conferences (Heidari et al., 2016). These guidelines provide researchers and authors with a tool to standardise sex and gender reporting in scientific publications, whenever appropriate. These guidelines encourage researchers to consider sex and gender differences in research design, study implementation, and scientific reporting, as well as in general science communication, where relevant. A review of published reporting guidelines to determine whether sex and gender were included revealed low use and integration of sex and gender concepts, plus incorrect use of these terms (sex and gender) (Gogovor et al., 2021). Of 407 reporting guidelines considered, only one reporting guideline met their standard for correct use of sex and gender criteria. Requirements to recognise the importance of sex differences is now becoming more widespread with a subset of the Nature Portfolio of journals requiring authors to describe whether, and how, sex and gender are considered in study design (Accounting for sex and gender, 2020). If no sex and gender analyses were carried out, authors will need to clarify why. This will apply to work with human participants, as well as other vertebrate animals and cell experimental studies. Although not specified (yet), presentation of *in silico* modelling work should also consider sex differences. This should mean that in the same way that ethics approval, clinical trials registration, or informed consent must be demonstrated where relevant, so too will consideration of sex and gender. Authors writing for Nature journals will also need to present “data disaggregated by sex and gender” where relevant. This means that rather than the more common approach of lumping male and female data together, it will need to be separated. This is a necessary move towards unravelling differences between males and females.

Even national funding bodies have begun to include policies to integrate sex, gender, and, more recently, diversity analysis into the grant proposal process, where these factors have been shown to play a role (Hunt et al., 2022). One example is the European Commission Horizon Europe Programme who are the largest funder to require sex and gender analyses, as well as analyses of other aspects of inclusion and how they interact, or intersect, known as intersectionality, in research design (European Commission, 2023).

When developing and parameterising models, experimental and clinical data is imperative. With respect to ensuring sex-differences are adequately captured in models, we must ensure that the data that we use is collected and analysed in a way that enables us to use sex as a biological variable. We need to ensure we are integrating sex (and gender) where relevant into our work to spark discovery, innovation and improve health impact. Echoing Nature’s wise words “Accounting for sex and gender makes for better science” (Accounting for sex and gender, 2020).
